# Is laccase from medicinal mushroom *Cerrena unicolor* cytotoxic to colon cancer cell line CT-26?

**DOI:** 10.1371/journal.pone.0322211

**Published:** 2025-05-08

**Authors:** Daria Sondej, Dominika Pigoń-Zając, Magdalena Jaszek, Dawid Stefaniuk, Anna Matuszewska, Kamil Bielak, Grzegorz Opielak, Teresa Małecka-Massalska, Mansur Rahnama-Hezavah, Monika Prendecka-Wróbel

**Affiliations:** 1 Department of Human Physiology of the Chair of Preclinical Sciences, Medical University of Lublin, Lublin, Poland; 2 Department of Biochemistry and Biotechnology, Institute of Biological Sciences, Maria Curie-Skłodowska University, Lublin, Poland; 3 Student Science Club by Oncological Surgery Clinic, Medical University of Lublin, Lublin, Poland,; 4 Department of Dental Surgery, Medical University of Lublin, Lublin, Poland; ENCB-IPN: Instituto Politecnico Nacional Escuela Nacional de Ciencias Biologicas, MEXICO

## Abstract

**Introduction and objective:**

Colorectal cancer takes an increasing toll every year. Despite the dynamic development of pharmacology, there is still no drug that would be strong enough to cause apoptosis of cancer cells, but at the same time would be free from numerous side effects. Taking traditional Eastern medicine into account, studies were carried out using an extract - laccase (LAC) from a medical mushroom called *Cerrena unicolor*- on CT-26 colon cancer cells. Preliminary cytotoxicity tests have already confirmed that the examined extract affects cancer cells and at the same time has no significant effect on L929 cells. The Electric Cell-substrate Impedance Sensing system (ECIS) and standard methods were used in this work. ECIS used in this study is an advanced *in vitro* impedance measuring system.

**Materials and methods:**

The CT-26 and L929 cells were treated by five different concentrations of the LAC preparation ranging from 0.025 to 250 μg/mL. Concentrations selected for the ECIS system assay were: 0.25;2.5 and 250 μg/mL. The default optimal frequencies in the ECIS system for Resistance (R) 4000Hz, Impedance (Z) 16000Hz, Capacitance (C) 64000Hz were used.

**Resluts:**

ECIS results demonstrate the potential anti-cancer activity of the laccase preparation against CT-26 cancer cells, and affect theL929 cells in to a lesser extent. Thanks to the use of the ECIS research technique, it was possible to monitor live changes in cell morphology and physiology, which translates into accurate conclusions about the action of the tested preparation.

## 1. Introduction

### 1.1. Laccase

Nowadays, there is a constantly growing interest in natural preparations. There is an increasing number of scientific reports on the promising effects of biological extracts, particularly those from medicinal mushrooms, on various types of cells. The effect of mushroom preparations on cancer cells is particularly interesting. This is a very important scientific problem due to the numerous and very unfavorable side effects for patients of currently used anticancer therapies.

Mushrooms are considered a good source of biologically active substances. In addition to their obvious culinary and nutritional values, they have anticancer, antibacterial, antiviral, and immunomodulatory effects [1].

It should be noted that in countries such as Russia, Japan, China and Korea, modern clinical medicine is also based on preparations obtained from mushrooms. Moreover, in almost 130 diseases these preparations are effective in possible therapeutic usage ware described. According to preclinical studies, such preparations seem to be relatively safe, also in long-term therapy. However, there are still few phase III studies conducted in small grups [2]. *Cerrena unicolor* is also called as “Turkey Tail” or “Mossy Maze Polypore”. It is a *Basidiomycetes* fungus that commonly occurson dead trunks of deciduous trees in the form of an arboreal hub. It is known that in some cultures it was used for medicinal purposes as an element of traditional folk medicine [3,4] for many years. It is important to note that the scientific evidence supporting its medicinal properties is still in the early stages, and more research is needed. The possible medical use of preparations obtained from this mushroom is very interesting. The list of positive effects of *C. unicolor* preparations includes, first, the immunomodulatory effect [5]. In close connection with the immunomodulatory effect of preparations obtained from *C. unicolor*, antioxidant [6] and anticancer [7] effects have also been described. It is important to emphasize that while these studies suggest potential medical applications, further research, including clinical trials, is needed to validate and establish the efficacy and safety of *C. unicolor* for specific medical conditions. As with any natural product, consulting with healthcare professionals is crucial before considering its use for medicinal purposes. Among the valuable substances that can be obtained from *C. unicolor* and have properties that predispose them to use in medicine, LAC is very promising. LAC is an enzyme that belongs to the multicopper oxidase family. In addition to LAC, the mushroom also provides other enzymes such as cellulases, xylanases, oxidases and terpene cyclases, which were obtained only a few years ago. It is sesquterpenoids that give the mushroom its characteristic floral and fruity scent [8].

It has gained attention due to its ability to catalyze the oxidation of a wide range of substrates, particularly phenolic compounds, with the reduction of molecular oxygen to water. This valuable enzyme obtained from *C. unicolor* has been repeatedly described in the scientific literature in various contexts [9–11]. It is worth the use of LAC in chemical and industrial processes such as bioremediation, the wood industry, paper pulp bleaching and the tanning industry [12].

*C. unicolor* is a mushroom with promising medical potential, hence the interest of numerous scientific centers in extracts or pure active proteins obtained from this mushroom, especially in the context of its anticancer effect. The preparations derived from *C. unicolor* and the fungus itself was studied and described for years. Various research centers have proven the positive effects of various preparations, extracts and mixtures of substances derived from this mushroom with very promising antioxidant, antiviral, antibacterial, immunomodulatory and anticancer properties.

As more and more attention is being paid to the problem of bacterial antibiotic resistance, in 2018 interesting results of *C. unicolor* extracts antibacterial activity have been published by the research of Sevindik, M. [13]. Also an anitivirial activity, which is best demonstrated by laccase LAC, has also been monitored for years [14]. Each year still, more new publications appear confirming the antioxidant effects of this medical mushroom [15]. It is also worth noting that antiparasitic activity, e.g., against *Rhabditis Nematodes*was reported [16].

Laccase (LAC) is a very interesting enzyme whose activity has been described in many works, therefore, in this study, an attempt was made to determine its anticancer potential towards colon cancer cells. An example of such action may be research indicating the antiproliferative effect of LAC on chronic myeloid leukemia and cervical cancer cells [7].

### 1.2. Electric cell substrate impedance sensing

In this project, an attempt was made to characterize the action of LAC on two types of cells: normal L929 and colorectal cancer CT-26. The technique used in the research is based on measurements of electrical parameters. ECIS is a research method first described by Giaever and Keese, which enables real-time monitoring of changes in electrical parameters during cell culture. Changes in resistance, impedance and capacitance values in the cellular environment allow to conclude about functional and morphological changes in the examined cells colonies. Classic methods that have been carried out on cell lines for years provide insight into one parameter, such as the number, morphology or phenotype of cells, but unfortunately it kills the cells at a given time point, which does not allow monitoring the culture dynamics over time. The ECIS system enables non-invasive tracking of changes in electrical parameters in real time, which provides a lot of valuable information.

The research system uses electrodes emitting low alternating current and electrodes measuring voltage change mounted on the bottom of a standard matrix. Impedance is measured according to Ohm’s law, Z = V/l. When cells attach to the matrix and electrodes, they act as insulators, increasing impedance. The current flow is reduced in such conditions depending on the number of cells covering the electrode, the shape and type of cells covering the electrode surface. The design of the ECIS measurement system includes: two electrodes (one is a small working electrode, and the other is a large counter electrode at the bottom of the culture plate) connected to the edge of the culture chip, connected to the recording system. The whole thing is placed in an incubator under constant conditions of 37 °C and an atmosphere of 5% CO_2_. Once the plates are inoculated with cells, they drift down and attach to the electrode surfaces, which then pass current directly into the bulk electrolyte due to the intrusion of the anchored cell membrane above the surface of the overall system [17].

**Fig 1:**
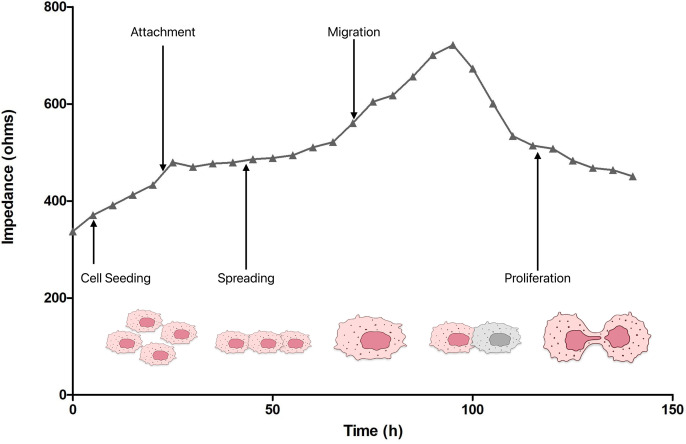
Impedance pattern of normal L929 cell line culture, showing corresponding cellular morphological changes.

By administering the tested preparation to the cell culture or stimulating the cells in another way, for example, by electrically irritating with a higher current frequency or mechanically irritating, or even by not ensuring the stability of the environment, changes can be brought about throughout the research system, which begins to register changes in impedance, resistance, and capacitance, which at the initial stage are the result of changes in the physical conditions of the culture. Then, changes in the values of electrical parameters were analyzed, which reflect changes in the cells themselves and their behavior. This, in turn, is reflected in physical parameters. In this system one of the most important factors is frequency. At low frequencies (<2000 Hz), most of the current flows between cells. At high frequencies (>40,000 Hz), most of the current flows directly through insulated cell membranes. Impedance at high frequencies is more influenced by the cell membrane, while at low frequencies the response is more dependent on the space beneath and between the cells [18]. Resistance (R) measurements are performed at low frequency, which allows for insight into the analysis of cell adhesion to the substrate. Therefore, monolayer formation was observed at a frequency of 4000 Hz. Based further on the analysis of Ebrahim et al., the parameter of 32,000 Hz was used as optimal for the impedance (Z), because at this value the monolayer begins to merge with the electrode. In turn, the value of 64,000 Hz was used to measure capacitance (C) when the cells became confluent [19].Confluence is directly related to migration, which is of fundamental importance in various physiological processes, such as wound healing, development, and immune response. On the other hand, migration is also of fundamental importance in pathophysiological processes, especially in the pathogenesis of cancer, because it is the migration and invasion of cancer cells that determine the formation of a secondary tumor. And the invasion process itself would not exist if migration did not exist. The basic definition of migration *in vitro* is cellular movement. As Fig 1 shows, depending on the frequency of the current flowing during the ECIS experiment, cells show different behaviors. The figure shows the growth characteristics of untreated cells. Migrating cells are characterized by cytoskeletal rearrangements, changes in cell shape and cell polarization. Typically, migration is observed using a microscope in specific time units. However, for a detailed description of migration kinetics, it is the continuous monitoring that ECIS provides that is the future [20]. Capacitance provides an overall measure of electrode coverage by a layer of cells. Therefore, different way of acting of cells after inoculation of the substrate, adhesion, proliferation, and their response to factors added to the substrate result in a change in impedance [21]. It is known that impedance (Z) measured in real time reflects cell motility and the speed of reaching confluence. In turn, impedance (Z) can then be divided into resistance and capacitance so that it is possible to distinguish between adhesion and proliferation, which in turn makes ECIS superior to traditional methods that have limitations in this regard. As the cells reach confluency at the electrodes in the graph, a flattening of the slope associated with the capacitance (C) curve can be noticed. Therefore the capacitance (C) measurements reflect the coverage and the resistance (R) data represent the moment when a mature barrier is already formed [19]. Consequently, it is obvious to use the ECIS system to study the complex processes of multiplication, adhesion to the substrate, and cell regeneration of two different types of L929 and CT-26 cells under the influence of a very interesting enzyme, LAC. Laccase (LAC) is an enzyme gaining increasing interest as a potentially useful with anticancer and antiviral activity [14]. The so-called white rot fungi are very efficient producers of this enzyme.

This study presents the effect of laccase from the medicinal mushroom *C. unicolor* on the viability of colorectal cancer cells (CT-26) in the context of normal mouse fibroblasts (L929). The experiments were carried out using the ECIS measurement system and show changes in cells behavior depending on the concentration of the added substance or frequency of the applied current.

## 2. Materials and methods

### 2.1. Fungi culture conditions

*C. unicolor* (Bull. ex Fr. Murr.) was obtained from the culture collection of the Regensburg University and deposited in the fungal collection of the Department of Biochemistry and Biotechnology (Instutute of Biological Sciences, Maria Curie-Skłodowska University, Poland) under strain number 139 (ITS sequence deposited in GenBank under accession number DQ056858) [22]. Culturing conditions were carried out in a 2.5 L Bioflo III fermenter (New Brunswick Scientific, New Brunswick, NJ, USA) which were containing 2 L of a sterilized Lindenberg and Holm medium, at 28 °C [23]. The medium was inoculated with 10-day old homogenized fungal mycelium (10% of total volume), aerated at 1 L of air per minute, and stirred at 100 rpm. Antifoam B emulsion (Sigma, St. Louis, MO, USA) was occasionally added to the fermenter cultures to break the foam. Incubation was carried out until the idiophase begins. The idiophase was determined following Jennings and Lysek’s recommendations [24].

### 2.2. Preparation of the Laccase Fraction (LAC)

After preliminary analysis including comparison of the results of studies of the effect of the preparation on other types of cancer, experiments were started from September 23 and completed in January 2024.The isolation, preparation, and characterization of LAC was conducted accordingly to the procedures described previously by Jaszek et al. [25]. As previously mentioned, 10-day-old idiophasic cultures utilized for the isolation of specific subfractions were filtered through Miracloth (Calbiochem) and washed with MQ water [23]. The culture liquid, after mycelium separation, was centrifuged at 10,000 × g for 15 minutes. The residual culture medium was separated into two fractions using an Ultracel tiny cartridge (10 kD cut-off) and an ultrafiltration system (Pellicon 2 tiny holder, Millipore, Bedford, MA). The solution containing components over 10 kDa was used as a source of LAC, which was purified using the previously reported method [26].

LAC isolation and detection were carried out as previously described [25]. The concentrated protein fractions were subdivided by anion exchange chromatography on a DEAE Sepharose column (2.5 × 15 cm) using an FPLC system (Bio-Rad, Richmond, USA) equilibrated before separation with 20 mM Tris-HCl buffer (pH 6.5). The appropriate protein pool was eluted using a linear gradient of NaCl (0.1–0.5 M) at a flow rate of 1 mL/min for 360 minutes and monitored at 280 nm. LAC activity fractions were collected and desalted on a Sephadex G-50 column (5.0 × 20 cm). Throughout the purification process, the temperature was maintained at 4 °C. The LAC isoform solution was lyophilized using the Freezone 12 Freeze Dry System (Labconco, Kansas City, USA) [27]. The activity of LAC was measured by oxidizing 0.025 mM syringaldazine (4-hydroxy-3,5-dimetoxybenzaldehydeazine) in 50 mM buffer at pH 5.3 [28]. As it was described in the previous publication, 1 mg of a lyophilized LAC isoform combination mixed in 1 mL of MQ water with activity of 1 150 110 nkat/L (specific activity 3495.4 nkat/mg of protein) was employed in this experiments [14,25].

### 2.3. Culture conditions

Tests were conducted on two cell lines. The first examined cells were mouse fibroblast cell line – NCTC clone 929 (L cell, L929, derivative of Strain L; ATCC® CCL-1™). The second one was colon carcinoma culture CT-26 (N-nitroso-N-methylurethane-(NNMU)-induced, undifferentiated colon carcinoma cell line, cloned to generate the cell line designated CT26.WT; ATCC® CRL-2638™). Both cell lines were cultured accordingly to the manuals. The L929 cell line was cultured in complete MEM Eagle (Pan-Biotech; Cat. No.: P04-08500) and CT-26 cells in RPMI-1640 Medium (Sigma-Aldrich, St. Louis, MO, USA; Cat. No.: R0883). Both mediums respectively, supplemented with 10% Fetal Bovine Serum (Heat Inactivated; Pan-Biotech; Cat. No.: P30-19375) and mix of antibiotics (100 IU/mL penicillin, 10 mg/mL streptomycin, and 25 g/mL amphotericin B; Pan-Biotech, Aidenbach, Germany; Cat. No.: P06-07300). Cultures were grown in a Galaxy 170R incubator with a constant temperature of 37 °C and 5% CO_2_ and relative humidity of 95% air. When the culture reached at confluence, the next stage of the examination was incubating the cells with LAC in different concentrations.

### 2.4. The cell viability evaluation – MTT assay

The assessment of the effect of LAC at different concentrations on the cell’s viability was carried out using the MTT assay. This test is based on the ability of the mitochondrial dehydrogenase enzyme to convert the tetrazole salt 3-(4,5-dimethylthiazol-2-yl)-2,5-diphenyltetrazolium bromide (Roche, Basel, Switzerland; Cat. No.: 11465007001). The effects were observed immediately as the soluble orange tetrazolium salt was converted to a dark blue precipitate - formazan - which was the product of the above reaction. It is amount corresponds to the number of living cells in the test sample [29]. L929 and CT-26 cells were seeded on 96-well microplate (Nunc) at a density of 1 × 10^4^ cells/well and left for 24 h. The next day, the culture medium was removed, and the cells were exposed to serial dilutions (0.025, 0.25, 2.5, 25 and 250 μg/mL of LAC made in a serum-free medium for 48 h and 72 h. Additionally the L929 cell line was exposed to rotenone at the following concentrations: 0.1, 1, 10, 100 nM, 1, and 10 μM. In the next stage, the tetrazolium salt solution was added to the wells and incubated for 4 hours. The formazan crystals were subsequently dissolved using 200 μL of DMSO, resulting in a colored solution, the intensity of which is measured by spectrophotometry at a 570 nm wavelength.

The experiment involved three independent repeats, in the same conditions.

### 2.5. The cell proliferation assay – BrdU assay

Another test performed was the BrdU test, which consists in the quantitative measurement of DNA synthesis. Bromodeoxyuridine (BrdU) is a synthetic thymidine analogue. As an analog, it is incorporated into the strand of newly formed DNA during its replication in the S phase of the cycle. The embedded molecules are then detected using specific enzyme-conjugated antibodies directed against BrdU. This reaction of the chromogenic substrate with the above enzyme allows the colorimetric analysis of the proliferative capacity of the cell. The BrdU Cell Proliferation Assay (Merck KGaA, Darmstadt, Germany; Cat. No.:2752) was performed according to the manufacturer’s instructions. Cells were seeded on 96-well microplate (Nunc) at a density of 2 x 10^5^ cells/mL in 100 μL/well of appropriate cell culture media and left for 24 h. After 24 hours of incubation the cells were exposed to serial dilutions (0.025, 0.25, 2.5, 25 and 250 μg/mL of LAC within 24–72 h. Properly prepared plates were measured spectrophotometrically at a wavelength of 450 nm [30]. The results were reported as an IC_50_, which is the concentration of a substance that suppresses tumor cell multiplication by 50% relative to untreated control cells.

### 2.6. The electric cell-substrate impedance sensing

**Fig 2:**
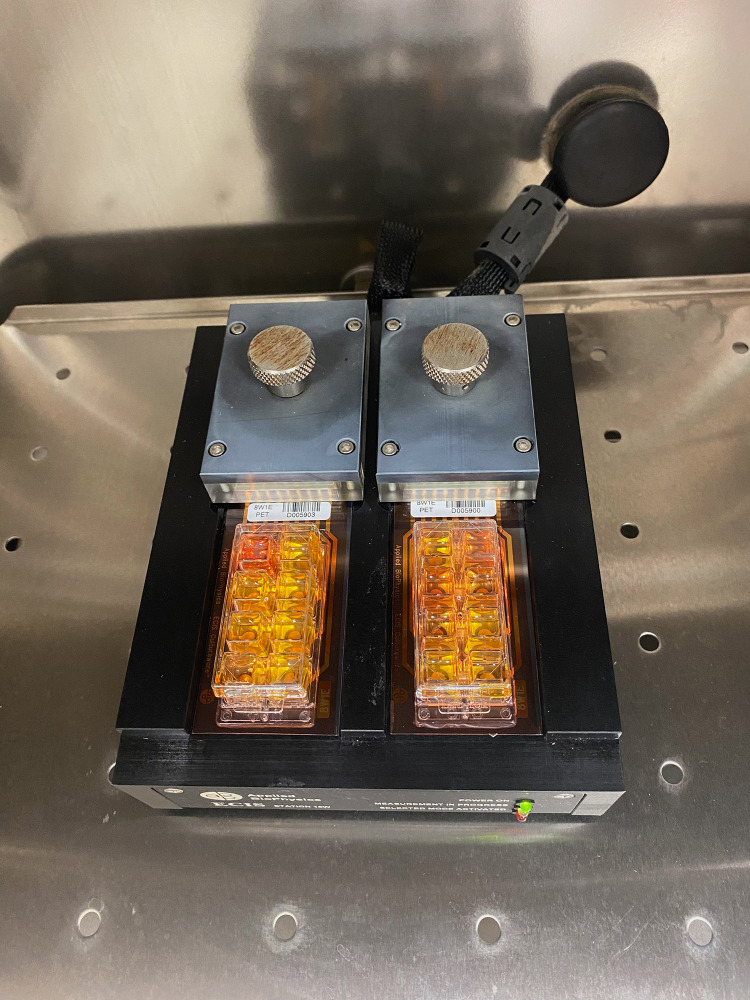
ECIS electrode plates in a docking station, placed in an incubator in the laboratory of Department of Human Physiology, Medical University of Lublin.

The ECIS system (Applied Biophysics, Inc. Troy, NY, USA) was used to measure impedance, capacitance and resistance. The system consisted of two units: a control station (Zθ) outside the incubator and a docking station with two 8-well plates within (Fig 2). The size of each well was approximately 0.8 cm^2^ of surface area for cell adhesion and development and capacity up to 600 µL. The electrodes used were 8W10E (Applied Biophysics Ltd., Troy, NJ, USA), with 10 active electrodes per well.

ECIS electrodes in a holder plate we located in a humid incubator at 37 °C and 5% CO_2_. The arrays were incubated with Eagle MEM (L929) and RPMI (CT-26) in the Galaxy 170R incubator for 24 hours prior to inoculation. After stabilization, the array was taken from the array station and seeded with cells. Arrays were inoculated with 600 microliters of cell suspension (1.2 × 10^5^ cells/mL) in each well. After 24 hours LAC was added to inoculated wells of the final concentrations: 0.25, 2.5 and 250 μg/mL in duplicate. Additionally, the L929 cell line was exposed to rotenone at the following concentrations: 0.1, 1, 10, 100 nM, 1, and 10 μM. The measurement was carried up to 120 hours. The maximum response for Z, R, and C occurred at different frequencies. In this study, the default optimal frequencies were used: resistance (R) 4000 Hz, impedance (Z) 32,000 Hz, and capacitance (C) 64,000 Hz. The ECIS system evaluated electrical parameters values after cell stimulation with the tested agents to determine the resulting morphological changes. All steps were performed in accordance with the manufacturer’s recommendations.

### 2.7. ECIS wound assay

The ECIS (ECIS Zθ, Applied Biophysics, Inc. Troy, NY, USA) and 8W1E arrays were used to monitor L929 and CT-26 cells migrationduring an electrical wound assay. 8W1E arrays (Applied Biophysics Ltd., Troy, NJ, USA), with 1 active electrode per well and 250 μm diameter, were prepared and incubated as described above. Arrays were inoculated with 600 microliters of cell suspension (1.2 × 10^5^ cells/mL) L929 and CT-26 lines and placed in a humidified cell culture incubator at 37 °C and 5% CO_2_. The wound assayinvolved exposing confluent cultures after 24 hours by using the integrated electrical field module (40-kHz,1600μA, 30 s) for fibroblasts and (60-kHz, 1400 μA, 30s) for colon cancer cells. The medium was substituted with the various medium types, including 10% FBS as a positive control and 0.25, 2.5, 250 μg/mL LAC concentrations in duplicate.

The changes in electrical impedance (Z 32,000 Hz) were monitored in real time for the subsequent above 100 hours.

### 2.8. Statistical analysis

For statistical analysis, a one-way ANOVA test was performed using GraphPad Prism 5 (GraphPad Software Inc., San Diego, CA, USA), followed by Dunnett’s multiple comparison analysis. Data was analyzed with a significance level of p < 0.05 and presented as mean ± SD.The ANOVA test for analysis of variance was chosen due to the abundance of concentrations used in several tests [31]. Results were calculated using GraphPad Prism.

## 3. Results

### 3.1. The MTT results

**Fig 3:**
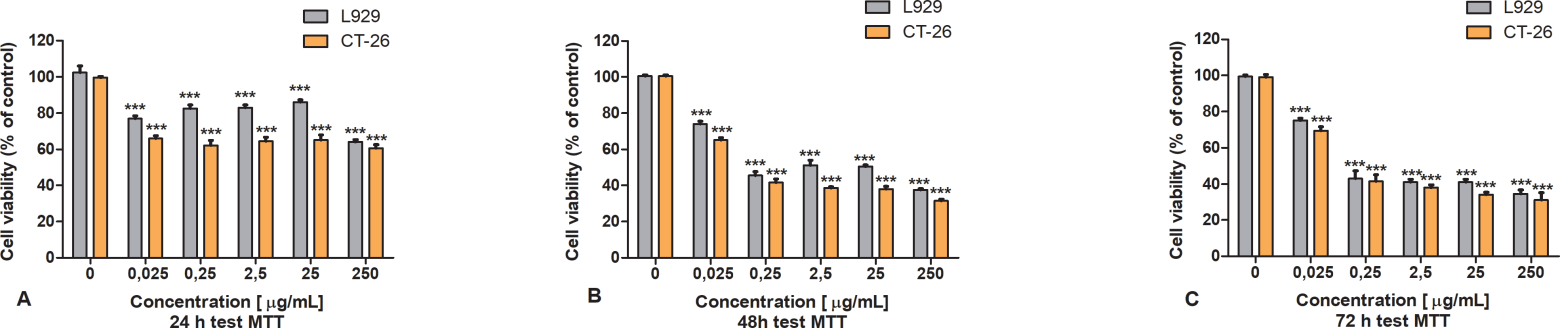
The effect of laccase on L929 and CT-26 cell viability measured by MTT assay; Data are presented as mean value ± SDp: * P ≤ 0.05 ** P ≤ 0.01 *** P ≤ 0.001one-way ANOVA, Dunnett’s test.

To determine the potential cytotoxic effect of the LAC preparation on CT-26 and L929 cells, the MTT test was performed as the first stage of the study. In Fig 3, the decrease in viability compared to normal cells was presented after after 48 h of incubation with LAC inCT-26 cultures. After 72 hours of incubation (Fig 3 C), viability does not decrease further. In the case of normal L929 cells relatively better viability was noted. In addition, a dose effect may also be noted. It can be seen here that with the increase in laccase concentration, the viability of the tested cells decreases.

### 3.2. The BrdUResults

**Fig 4:**
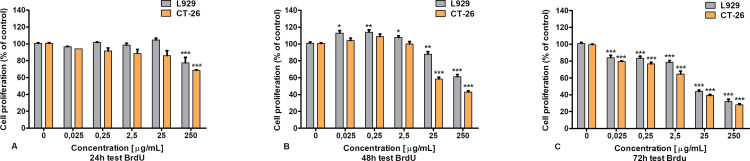
The BrdU assay of an influence of laccase on L929 and CT-26 cells; Data are presented as mean value ± SD p: * P ≤ 0.05 ** P ≤ 0.01 *** P ≤ 0.001one-way ANOVA, Dunnett’s test.

By the use of cell proliferation test it was tested if LAC from *Cerrena unicolor* affects the level of cell proliferation, in both cell lines. The greatest decrease in proliferation was shown inCT-26 cultures after 72 hours of incubation with preparation, in nearly all concentrations. As shown in Fig 4 the higher the concentration, the greater the decrease in the tested parameter. However, the greatest difference between CT-26 and L929 cell proliferation levels was noted after 48 hours of treatment for doses of 25 and 250 µg/ml.

The IC_50_ LAC results for both cell types are shown in Table 1. The preparation had a more effective antiproliferative impact against CT-26 tumor cells (IC_50_ = 3.849 µg/mL) compared to L929 cells (IC_50_ = 5.857 µg/mL).

**Table 1:** IC_50_ values of LAC on CT-26 and L929 cell lines as determined during 72h period of BrdU assay, using non-linear, four-parameter regression analysis.

Cell line	IC_50_ µg/mL
CT-26	3.849
L929	5.857

### 3.3. The ECIS results

Based on the performed viability and proliferation tests, three concentrations of the preparation were selected at which the differences in the effect on the cells could be best visualized. In the following stage, 0,25µg/mL, 2,5 µg/mL and 250 µg/mL concentrations of the preparation were administered to CT-26 and L929 cultures in the ECIS system, and the effect of LAC on both cell lines was monitored per 120 hours.

**Fig 5:**
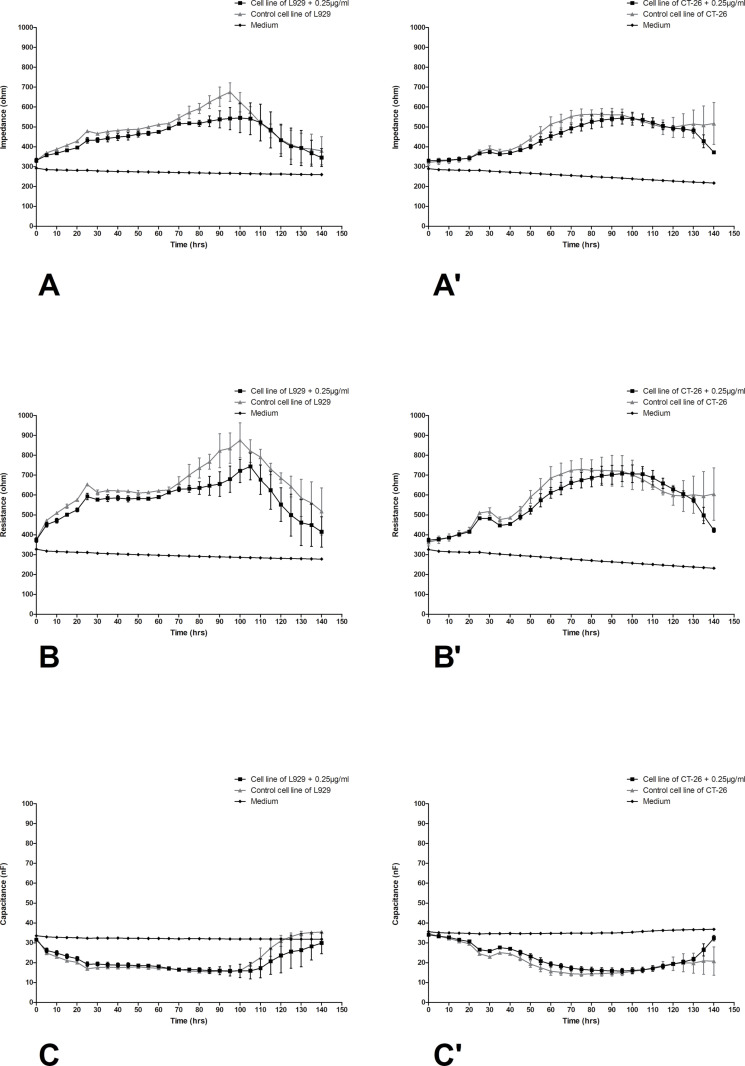
Impedance, resistance, and capacitance monitoring of the cell lines L929 (A–C) and CT-26 (A’–C’) during 80h treatment with LAC in concentration 0.25 µg/mL. Data are presented as mean value ± SEM.

**Fig 6:**
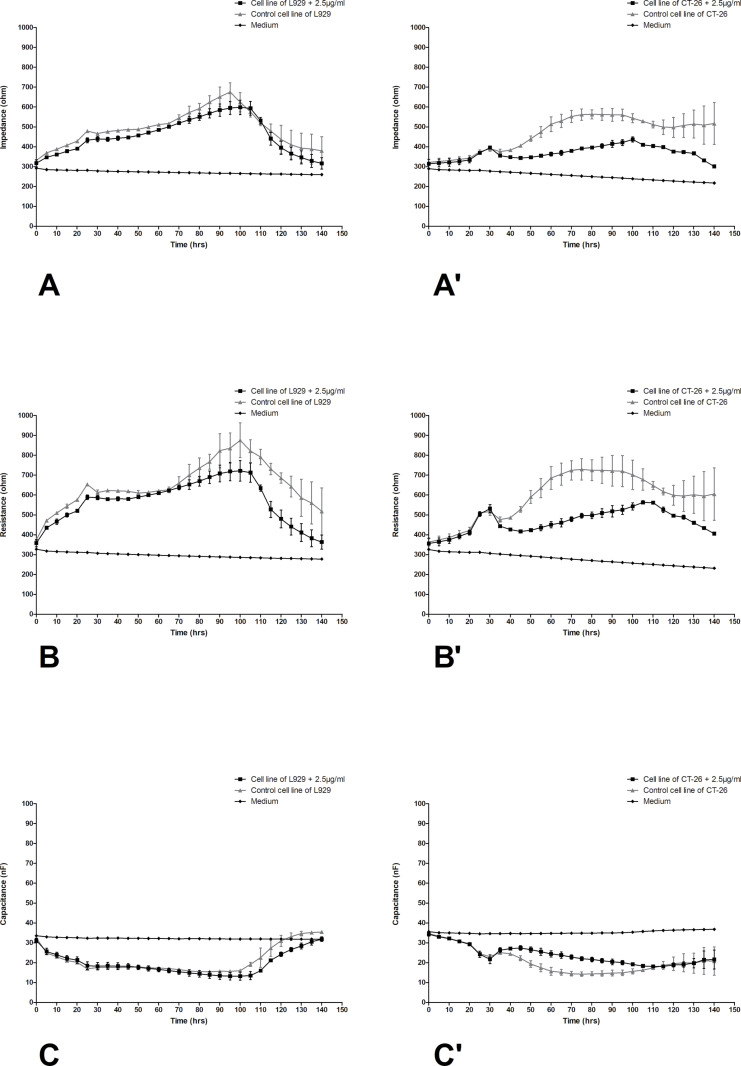
Impedance, resistance, and capacitance monitoring of the cell lines L929 (A–C) and CT-26 (A’–C’) treatment with LAC in concentration 2.5 µg/mL. Data are presented as mean value ± SEM.

**Fig 7:**
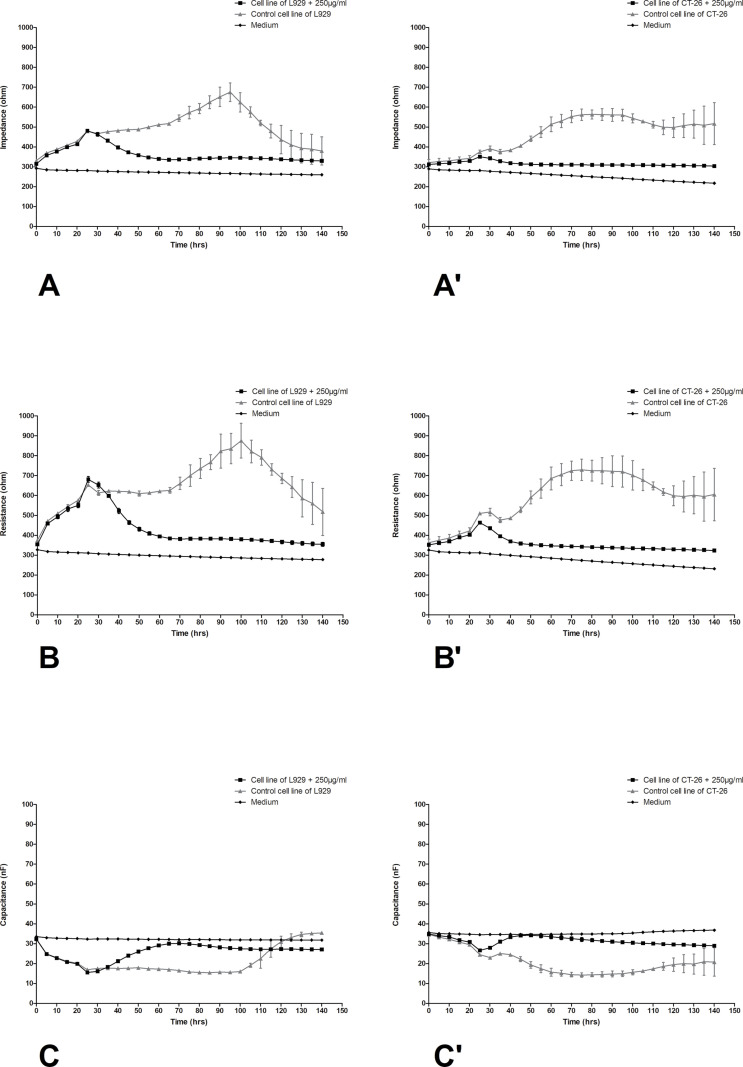
Impedance, resistance, and capacitance monitoring of the cell lines L929 (A–C) and CT-26 (A’–C’) treatment with LAC in concentration250µg/mL. Data are presented as mean value ± SEM.

The differences in impedance, resistance, and capacitance were observed in both types of cells, however, significant changes ware noticed in cancer cells. In Fig 5 changes in impedance, resistance and capacitance in L929 (A-C) and CT-26 (A’-C’) cell cultures treated with the preparation at a concentration of 0.25 µg/mL were presented. Based on the obtained numerical data, it can be concluded that the addition of the lowest selected LAC concentration only slightly reduced the impedance and resistance in the research system with fibroblast cells to values of approximately 500 and 700 ohms, respectively. Similarly, in the case of colorectal cancer cells, the decreases in impedance and resistance are trace, practically not changing the course of cell growth, remaining at a level between 400 and 700 ohms for both control cultures and laccase-treated cells. Fig 6 shows changes in the values of electrical parameters in L929 (Fig 6A–C) and CT-26 (Fig 6A’–C’) cultures after the application of LAC at a concentration of 2.5 µg/mL. In this variant, significant changes in electrical parameters were observed, especially in the case of CT-26 cells. In this variant of the culture of normal L929 cells, the tested preparation had only a slight effect on the cells. A decrease in impedance can be observed from a maximum value of approximately 700 ohms in the 90th hour of the experiment to a value lower by 100 ohms. In the case of resistance, the changes are slightly larger, but still within the range of permissible changes, while the almost identical and stable capacitance values only confirm that LAC at a concentration of 2.5 µg/mL does not disturb the stability of the normal fibroblast culture. The changes in all tested parameters in the CT-26 culture treated with LAC at a concentration of 2.5 are very significant. The greatest decreases in the impedance of CT-26 cells were observed between 50 and 140 hours of culture. For cancer control cells, the impedance values remained at the level of approximately 600 ohms in the mentioned time range, while in the case of CT-26 cells treated with LAC at a concentration of 2.5µg/mL, the impedance values were significantly lower because they reached a height of approximately 400 ohms. The cytotoxic effect of this LAC concentration is also confirmed by the capacitance values (Fig 6C’). The capacitance value in this case increased by approximately 10 nF between the 50^th^ and 100^th^ hour of culture. The trend is confirmed by the results obtained in the case of a variant of the experiment involving the action of the preparation at a much higher concentration, i.e., 250, on both types of cells. These values are illustrated in Fig 7. In this variant, the tested preparation had a very significant effect on both types of cells. As in the case of L929 cells, where impedance drops to 350 ohms were achieved, similarly, drops in impedance values for cancer cells were achieved under the same conditions. Similarly, the resistance and capacitance values confirm the cytotoxic effect of LAC on cells of both types, and a significant dose effect is also visible.

### 3.4. ECIS scratch assay results

**Fig 8:**
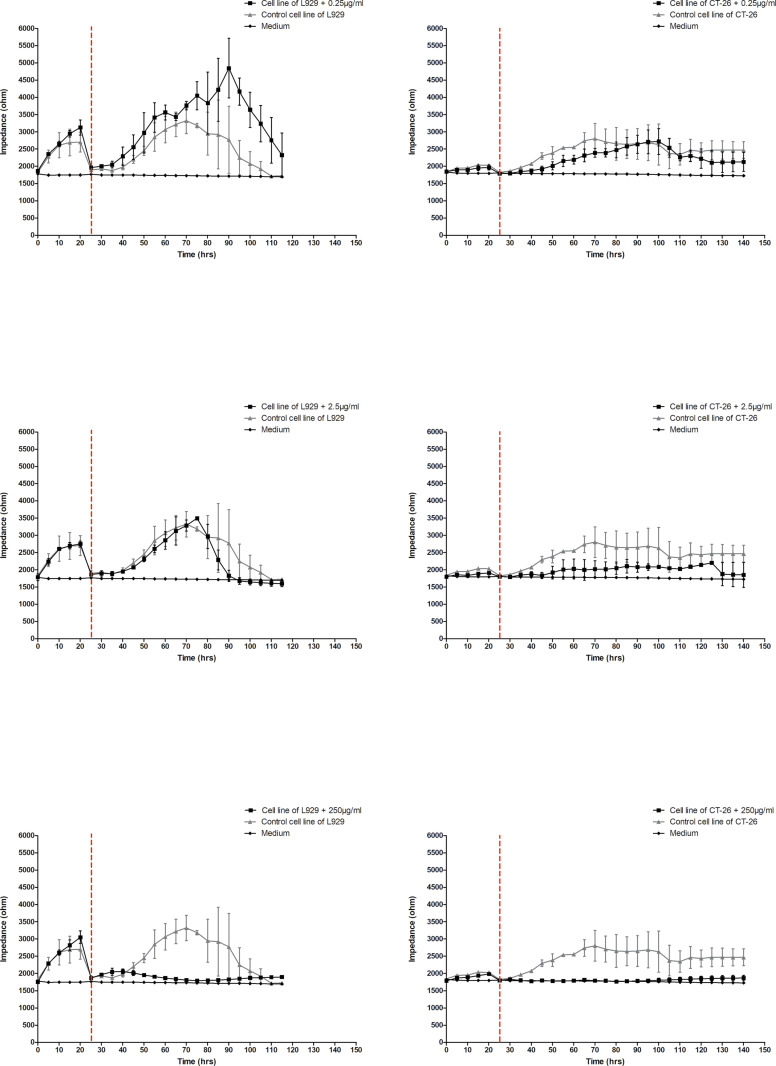
Effect of LAC on L929 cells and CT-26 cells in scratch assay in 3 concentrations. Data are presented as mean value ± SEM. The dashed line represents the moment the scratch was made.

The migration process and the possible effect of LAC on it was assessed next. The electrical signals were used to both create a precise scratch and monitor the process of cell infiltration, migration and healing. The scratch, which is created because of a short-term, controlled flow of high-value current used in the ECIS system, targets only a small population of cells in contact with an active electrode with a diameter of 250 micrometers, creating a precise scratch/wound with a diameter of 250 micrometers. In Fig 8 the results of the scratch test in a culture of L929 fibroblast cells after treatment with the LAC enzyme at three concentrations are presented. As can be seen, in the case of the lowest concentration of LAC used (0.25 µg/mL), the effect of the preparation was insignificant. Based on even a relatively significant increase in the impedance value, an increase in the viability and migration of cells treated with LAC can be concluded. This is evidenced by the increase in the impedance value to 4000 ohms in the 50–70-hour interval of the experiment, i.e., approximately 20 hours after the scratch/wound occurred. At the same time, in the control variant, the impedance values were 2000–3000 ohms. The middle concentration of the preparation (2.5 µg/mL) had a strong effect on L929 cells. In the first case, LAC caused a weakening of the growth and migration potential, because obtained impedance values reached a maximum of approximately 3000 ohms in the 50^th^ hour of culture, i.e., approximately 25 hours from the formation of the scratch, after which the cells began to die, as evidenced by a gradual decrease in the values of impedance. However, in the case of control cells, after scratching, the impedance values gradually increased up to 3500 ohms in the 70^th^ hour of culture and began to gradually decrease only after 90 hours. The highest concentration (250µg/mL) had the strongest effect on fibroblasts, practically inhibiting their growth and migration.

A significant effect of the preparation for colorectal cancer cells was found. As in the case of fibroblast cells, the highest concentration (250 µg/mL) affected cells most. In this variant, the impedance values for CT-26 cells treated with LAC did not increase after scratching, which indicates a strong inhibitory effect of LAC at a concentration of 250µg/mL on the growth and migration of colorectal cancer cells. Under the same conditions, cells not treated with the preparation were characterized by relatively stable growth and migration potential.

### 3.5. Rotenone results

**Fig 9:**
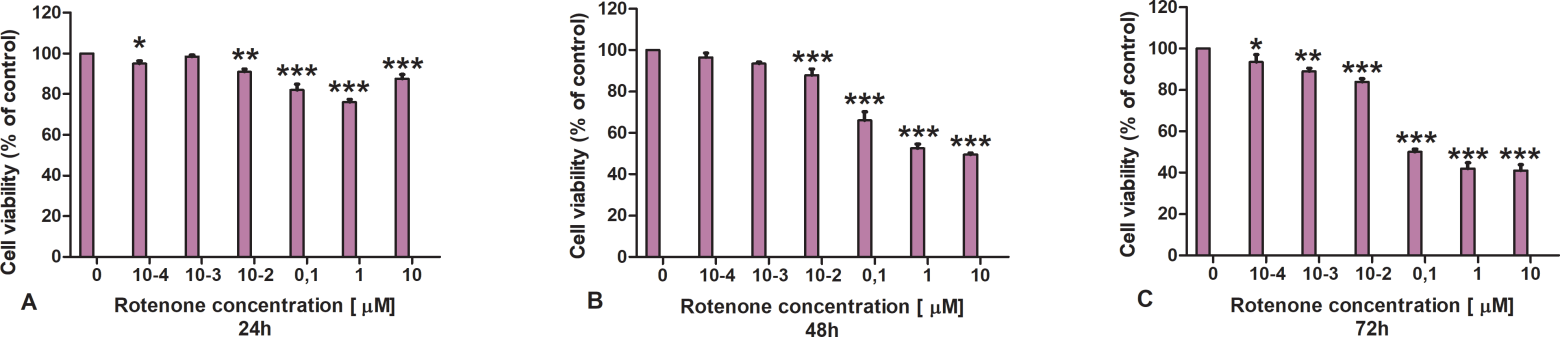
Effect of rotenone on the L929 cell line assessed by the MTT assay. Data are presented as mean value ± SD; p: * P ≤ 0.05 ** P ≤ 0.01 *** P ≤ 0.001 one-way ANOVA, Dunnett’s test.

**Fig 10:**
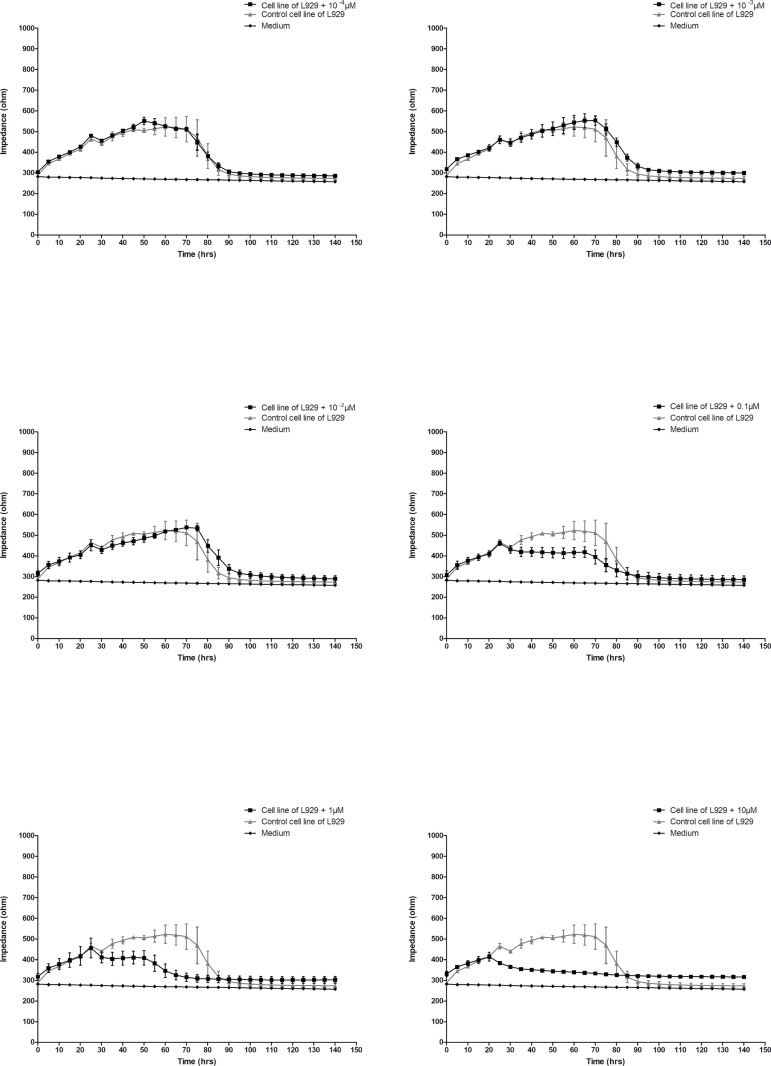
Effect of rotenone on the L929 cell line monitored by the ECIS method. Data are presented as mean value ± SEM.

To demonstrate the effect of a known and well described cytotoxic substance, rotenone was used, and its effect was examined on L929 cells. Many studies have already indicated rotenone as an apoptosis-inducing substance; therefore it is a reference for confirmed cytotoxic effects [32]. In the first stage of this experiment, a standard MTT test was used Fig 9. Based on the results obtained, it can be concluded that the cytotoxic effect of rotenone can be noted only after 48 hours at concentrations from 0.1 nM to 10 μM and after 72 hours also at concentrations of 0.1 nM-10 μM. To determine more precisely how rotenone affects the viability of normal cells, an ECIS experiment was performed (Fig 10). Based on the assessment of impedance changes, a clear cytotoxic effect of rotenone on normal L929 cells can be concluded at the three highest concentrations. The differences in impedance values between control cells and cells treated with rotenone were approximately 100 ohms at concentrations 0.1 nM and 1μM and over 200 ohms at concentration 10 μM in the time interval from 20 to 80 hours of culture in all variants. From the 80th hour of culture, the impedance decreased to the impedance value of the culture medium, which indicates cell death.

## 4. Discussion

Laccases from *C. unicolor* and other fungi gained attention for their ability to catalyze reactions with diverse substrates, making them valuable enzymes for biotechnological applications. Nevertheless, in the context of this research, the therapeutic potential of LAC is most important. Interest in LAC and its medical potential has become increasingly scientifically significant. Recently Kudanga et al., described the use of laccases in the synthesis of bioactive compounds, highlighting their potential in the pharmaceutical and fine chemical industries [33]. In turn, the research published by Mizerska-Dudka et al. demonstrated several activities of LAC from *C. unicolor*, such as: antiviral, immunomodulators and anticancer [14]. They tested the antiviral, immunomodulatory, and anticancer activities of the enzyme. However, there is limited direct evidence supporting the anticancer effects of LAC from *C. unicolor.* The potential anticancer effects of laccases are not well-established, and most research on *C. unicolor* and cancer has focused on other bioactive compounds found in the fungus, such as polysaccharides and secondary metabolites. These components have shown some promise in preclinical studies, but more research is needed to understand their mechanisms and potential clinical applications. The study that explored the antioxidant and anticancer activities of an ethanolic extract from *C. unicolor* against human colon adenocarcinoma cells was published in by Vieira Gomes et al. [6]. It is also believed that LAC, like *G. lucidum*, may have the ability to scavenge free radicals, which may protect cells from oxidative stress. Reactive oxygen species can destroy cell DNA, which leads to many diseases. Therefore, enzymatic neutralization of ROS seems to be a very good remedy [25].This study focuses on the potential anticancer effect of LAC, exerted through the effect that this enzyme has on the functioning of colorectal cancer cells compared to the effect of LAC on normal fibroblast cells.

For many years, colorectal cancer has been considered the second most common cancer. Data from March 2023 indicate that approximately 1.7 million people are diagnosed with it annually. Over the years, the most effective method of treatment remains surgery, which involves severe recovery. Chemotherapy is also often used and has many side effects [34]. Furthermore, this tumor has an inconsistent pathophysiological picture, which makes it even more difficult to eradicate [35].

In addition to the standard methods used in such studies, i.e., MTT and BrdU assays, in experiments aimed at characterizing changes in selected cell cultures under the action of LAC, the ECIS technique was used. The MTT and BrdU tests were carried for the initial selection of concentrations due to their speed and low costs. After showing promising results in both of these tests, an ECIS study was conducted

The Electric Cell-Substrate Impedance Sensing (ECIS) method is a non-invasive and real-time technique used to monitor cellular behaviors, including cell adhesion, migration, and proliferation, by measuring changes in electrical impedance. It has been employed in anticancer research to study the effects of various compounds, or drugs.

ECIS provides valuable information on the dynamics of cellular responses, making it a useful tool in cancer-related studies Lee et al., investigated the effects of different extracts of Chaga mushroom on the antitumor activity in HT-29 human colon cancer cells [36]. Also, in different study the anticancer effects of *Agaricusblazei* on pancreatic cancer cells using various methods [37]. Particularly important for the development of medicine is a change in thinking about the precise diagnosis of pathology and therapy in context of using pharmacological agents that are safer for the patient, and cause fewer side effects, remaining effective preparations. From this point of view, the method and effectiveness of techniques used in research to determine the healing potential of a given substance, compound, or mixture of substances is of particular importance in broadly understood biomedicine. Measurement of chosen electrical parameters, i.e., impedance, resistance, and capacitance of the cell membrane in real time can be an important complement to the results obtained by traditional analytic methods. The method based on monitoring changes in electrical parameters seems to be accurate and promising, for examining the behavior of cells as well as the condition of their entire populations, pioneered by Giaever and Keese [38]. The main advantage of ECIS technique is that it is non-invasive, unlike fluorescence, chemiluminescence or radioactive labeling methods often used in *in vitro* tests. Therefore, that the ECIS method could be a perfect complement to standard tests or an initial screening procedure [39].

After stimulation of cells with the tested laccase preparation or another stimulus (scratch test), changes in the cell population were observed, including changes in their viability, migration and proliferation potential. Only after some time does it begin to reflect changes in the structure of cells themselves and their behavior, what, is reflected in physical parameters. Measuring changes in electrical parameters, the condition of cells was assessed based on changes in impedance, resistance, and capacitance. Cells that die either due to the cytotoxic effect of the administrated substance or due to natural causes, depletion of nutrients and congeries of metabolites in the culture medium begin to detach from the culture medium, which changes the impedance value [40]. Considering the properties and condition of cell membranes, it should therefore be stated that in the ECIS measurement system, cells constitute a parallel connection (analogous to electrical systems) of a resistor and a capacitor. In this case, electrical resistance is the inverse of the current flow, while capacitance describes the separation of electrical currents in the membrane bilayer, which causes cell polarization. Capacitance therefore reflects the state of coverage of the electrodes by the cell layer [41]. In the presented work, the effect of the LAC enzyme from *C. unicolor* on cells was assessed. ECIS measurements used for monitoring cell proliferation, can be intended to determine factors in cells and culture conditions that affect the rate at which a monolayer of cells reaches confluence. As the number of cells increases, the surface area of the electrode covered with decomposed cells increases correspondingly, which causes an increase in impedance. These changes in impedance can be related to the relative rate of cell proliferation or, more precisely, the rate at which the substrate is occupied by cells [19,42]. Another process potentially subject to evaluation in the ECIS system is migration. Cell migration is an essential process that occurs early in development and maintains homeostasis from the beginning to the end of an organism’s life. Due to its importance, the migration process of both normal and cancer cells has become a leading research goal of this work [43].Traditional methods for assessing wound healing lack repeatability and precision and were widely criticized. Thanks to the analysis of changes in the electrical impedance value in cell-measuring matrix systems (ECIS), the wound healing test can now be significantly more precise, which makes the repeatability of the obtained results very high. On this basis, it was noticed that LAC from *C. unicolor* limits the migration of cancer cells, which is important when considering the future use of this enzyme. Analysis of the characteristics and physiological functions of individual cell types in the presence of the tested substances can be an effective way to understand many contemporary biological and biomedical problems. Routine analyzes carried out on cell lines allow, among others: to assess the number, morphology and phenotype of cells, their viability (proliferation, apoptosis), metabolic activity, synthesis of intracellular transcription factors and the release of several substances (cytokines, chemokines). Due to the above, attempt of better understanding of processes that may ultimately lead to the death of the examined cell or, stimulation of its proliferation seems to be reasonable. However, it’s important to note that research in this area is still in its early stages, and more studies are needed to fully understand and harness the medicinal potential of laccases.

## 5. Conclusion

The current research confirms that an enzyme from the medicinal mushroom *Cerrena unicolor* LAC has antiproliferative effects and a decrease in the viability of CT-26 colon cancer cells, while the viability of normal L929 cells decreases only slightly, which is confirmed by results obtained in ECIS research. Additionally, it was noted that LAC causes a decrease in impedance and resistance in CT-26 compared to normal cells. This is reflected by the lowered viability of CT-26 cells. In the study, the dynamics of electrical changes on the CT-26 and L929 lines were monitored using the ECIS method. Therefore, this method can be successfully used alone to assess the cytotoxicity of a substance, or as an additional method in combination with commonly used methods such as ELISA. Additionally, this method offers a chance for better understanding of microbiological processes taking place in cancer cells.
